# Dendrimeric Systems and Their Applications in Ocular Drug Delivery

**DOI:** 10.1155/2013/732340

**Published:** 2013-12-14

**Authors:** Burçin Yavuz, Sibel Bozdağ Pehlivan, Nurşen Ünlü

**Affiliations:** Pharmaceutical Technology Department, Faculty of Pharmacy, Hacettepe University, 06100 Ankara, Turkey

## Abstract

Ophthalmic drug delivery is one of the most attractive and challenging research area for pharmaceutical scientists and ophthalmologists. Absorption of an ophthalmic drug in conventional dosage forms is seriously limited by physiological conditions. The use of nonionic or ionic biodegradable polymers in aqueous solutions and colloidal dosage forms such as liposomes, nanoparticles, nanocapsules, microspheres, microcapsules, microemulsions, and dendrimers has been studied to overcome the problems mentioned above. Dendrimers are a new class of polymeric materials. The unique nanostructured architecture of dendrimers has been studied to examine their role in delivery of therapeutics and imaging agents. Dendrimers can enhance drug's water solubility, bioavailability, and biocompatibility and can be applied for different routes of drug administration successfully. Permeability enhancer properties of dendrimers were also reported. The use of dendrimers can also reduce toxicity versus activity and following an appropriate application route they allow the delivery of the drug to the targeted site and provide desired pharmacokinetic parameters. Therefore, dendrimeric drug delivery systems are of interest in ocular drug delivery. In this review, the limitations related to eye's unique structure, the advantages of dendrimers, and the potential applications of dendrimeric systems to ophthalmology including imaging, drug, peptide, and gene delivery will be discussed.

## 1. Introduction

Drug delivery to the eye is still one of the most challenging tasks for pharmaceutical scientists. The eye is characterized with its complex structure with high resistance to drugs as well as other foreign substances. The anterior and posterior segments of the eye function both independently upon an ocular application [1]. Thus, ocular drug delivery can be classified into anterior and posterior segments.

Conventional drug delivery systems are not effective enough to meet the requirements in the treatment of ocular diseases [2]. However, “more than 90% of the marketed ophthalmic formulations” are in the form of eye drops, and most of them target the “anterior segment eye diseases” [3]. Poor bioavailability of drugs from ocular dosage forms is mainly due to the “precorneal loss factors” including solution drainage, lacrimation, tear dynamics, tear dilution, tear turnover, conjunctival absorption, transient residence time in the cul-de-sac, and low permeability of the corneal epithelial membrane which are the major challenges to anterior segment drug delivery following topical administration.

Treatment of “posterior segment diseases” still remains as an unsolved issue. Most of the ophthalmic diseases affect neural retina, choroid, and vitreous. For example, glaucoma, diabetic retinopathy, age-related macular degeneration (AMD), and various forms of retinitis pigmentosa are damaging the posterior eye segment, which may cause impaired vision and even blindness [4]. Delivery of drugs to the posterior segment is more challenging than to the anterior segment, due to the acellular nature of the vitreous body and the longer diffusion distance [5]. Thus, posterior eye segment diseases have become an important therapeutic target with unmet medical needs. The major goal in the treatment of posterior segments diseases is the delivery of therapeutic doses of drugs to the tissues while reducing the effects. Systems developed to achieve this goal range from simple solutions to novel drug delivery systems, such as nanoparticles, liposomes, micelles, dendrimers, iontophoresis, and gene delivery systems [5–7].

Dendrimers are “tree-like,” nanostructured polymers that have been interesting in terms of ocular drug delivery. They are attractive systems for drug delivery due to their nanosize range, ability to display multiple surface groups that allows for targeting, and easy preparation and functionalization [8]. Ongoing studies in developing improved ocular dendrimeric systems may not only serve to enhance the drug delivery to the ocular surface, but also may provide effective delivery of therapeutic agents to intraocular tissues, such as the retina or choroid, using noninvasive delivery methods.

## 2. Challenges in Ocular Drug Delivery

Eye has a unique physical structure with protective barriers, which offers many challenges to the effective delivery of drugs to the eye. The eyeball is divided into 2 segments: the anterior segment containing the cornea, crystalline lens, iris, ciliary body, and fluid-filled aqueous humor and the posterior segment that includes the sclera, choroid vessels, retina, macula, optic nerve, and fluid-filled vitreous humor [2]. This organ is well protected with various specialized cellular modifications that give rise to various barriers that partially isolate the eye from the rest of the body, which can be a challenge for drug delivery [9]. These special processes/barriers are as follows.

- The “inner and outer blood–retinal barriers” separate the retina and the vitreous from the systemic circulation, and because of the absence of the cellular components in vitreous body, it reduces convection of molecules [10].
- The inner limiting membrane controls the exchange and entry of particles from the vitreous to the retina.
- The “blood-aqueous barrier” limits the transport of molecules from the blood to the inner part of the eye [11].
- Intact structure of corneal epithelium with desmosomes and tight junctions offers resistance to the passage of most drugs due to the presence of layers: hydrophobic epithelium, hydrophilic stroma, and hydrophobic endothelium [12].
- The tear film forms a mucoaqueous barrier that continuously washes away the particles at the anterior surface of the eye [9].

The anatomical and physiological barriers mentioned above are a challenge to ocular drug delivery. Solubility, lipophilicity, molecular size and shape, charge, and degree of ionization of the drug also affect the penetration rate to the eye [13]. Drug delivery systems' biocompatibility is also relevant when ocular delivery is concerned. The specific challenge of designing an ocular therapeutic system is to achieve an efficient concentration of the drug at the active site for the duration to provide the therapeutic efficacy [14, 15]. The requirements of an ideal topical formulation to the eye must be fulfilled as follows: the formulation must be well tolerated and easy to administer; avoid systemic absorption and increase drug retention time in the eye. Various ophthalmic formulations have been developed to improve ocular penetration, reduce toxicity, and improve tolerability [16, 17].

Typically “less than 5% of the topically applied drug” penetrates the cornea and reaches intraocular tissues, while most of the instilled dose is often absorbed systemically via the nasolacrimal duct and conjunctiva [3]. The eye drops are easy to instill but only a very small amount of the instilled dose is absorbed into the target tissues. It becomes necessary to apply large doses of drugs frequently to achieve the effective therapeutic dose which leads to an increase in both local and systemic side effects [18]. Since the penetration to cornea is often poor, either systemic or intravitreal administration (injection or implant) is required in order to treat posterior segment diseases. Due to strong blood-ocular tissue barrier, systemic administration requires large doses, while intravitreal injections and implants are very invasive and are associated with a high degree of retinal damage risk, such as retinal detachment and endophthalmitis [19]. Thus, there has been growing attention to transscleral route in order to deliver drug to the back of the eye [20]. Sclera is more permeable than cornea and even it is highly permeable to the large molecules of even protein size. However, it is more complicated to deliver the drug to retina, because in case of transscleral application the drug must pass across the choroid and retina pigment epithelium (RPE) [21].

The major goal is to develop suitable drug delivery systems with improved bioavailability of drugs, increased retention time, reduced side effects, cellular targeting, better patient compliance, and providing extended therapeutic effects [22]. Currently, nanocarrier-based ocular drug delivery systems including dendrimers appear to be the most promising way to meet the requirements of an ideal ocular drug delivery system.

## 3. Dendrimer Structure, Synthesis, and Properties

Dendrimers are monodisperse macromolecules with several reactive end groups that surround a small molecule and form an internal cavity. Their tree-like branched architecture displaying a variety of controlled terminal groups is in particular very promising for biomedical applications [23]. Especially low generation dendrimers can encapsulate hydrophobic drug molecules into their internal cavities. Because of this unique structure, dendrimers are able to solubilize poorly water-soluble drugs [24]. In addition to the extraordinary structural control, another outstanding feature of dendrimers is their actual mimicry of globular proteins. They are referred to as “artificial proteins,” based on their systematic, electrophoretic, dimensional length scaling and other biomimetic properties [25, 26].

**Figure 1:**
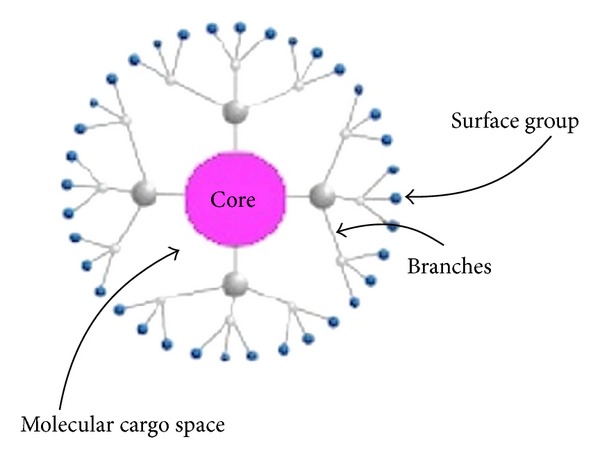
Schematic representation of a generation 2 dendrimer.

**Figure 2:**
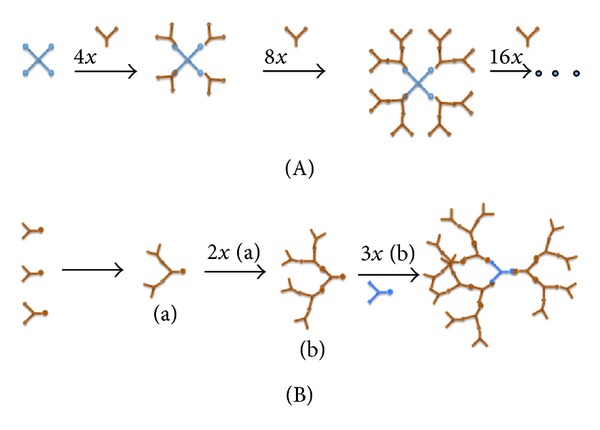
Schematic representation of divergent and convergent methods: (A) the divergent growth method (B) the convergent growth method.

Elements are added to dendrimer structure by a chemical reaction series and build a branching spherical structure from a starting atom such as nitrogen. The central core molecule should have at least two reactive functional groups and the repeated branches are organized in a series of “radically concentric layers” called ‘‘generations” [27]. A schematic representation of a generation 2 dendrimer is given in Figure 1. Dendrimers are generally prepared using either a divergent method or a convergent one [28]. In the *divergent method*, dendrimer grows outwards from a multifunctional core molecule. On the other hand, in the *convergent approach*, the dendrimer is constructed stepwise, starting from the end groups and progressing inwards. When the branched polymeric arms (dendrons) grew enough, they are attached to a multifunctional core molecule [29]. A schematic representation of divergent and convergent methods, is given in Figure 2. Other approaches have been developed based on the divergent and convergent methods such as *double exponential growth, lego chemistry, and click chemistry.* Preparation of monomers from a single starting material for both divergent and convergent methods is possible using double exponential growth approach. Then two result products are reacted to give a trimer, which can be used to repeat the growth again [30]. In lego chemistry strategy, phosphorus dendrimers are prepared from highly functionalized cores and branched monomers. After several variations in general synthetic scheme, a scheme is developed that allows multiplications of the number of terminal surface groups from “48 to 250” in one step [31].

Compared to other polymers, dendrimers have so many advantages such as their nanosize ranging from 1 to 100 nm with lower polydispersity index that allows them to avoid RES uptake. Furthermore, targeting anywhere in the body is also possible, thanks to the multiple functional groups on their surface which makes it possible to attach vector devices [32, 33]. Dendrimers have the ability to encapsulate drug molecules into their internal cavities which leads to enhanceed solubility, permeability, and retention effect depending on their molecular weight. It was reported that drug absorption is increased with dendrimers association in the cationic > uncharged > anionic order, where cationic dendrimers show permeation enhancement due to their ability of interacting lipid bilayers. Smaller generation dendrimers also have an enhancer effect on permeability since they have a better ability to move between cells [34].

Dendrimer cytotoxicity is related to the core chemistry; the nature of the dendrimer surface is the most influencing factor, because the interaction between surface cationic charge of dendrimers and negatively charged biological membranes is the main reason of toxicity. Lower generation dendrimers with anionic or neutral polar surface groups were reported to have higher biocompatibility as compared to higher generation dendrimers with neutral apolar and cationic surface groups. It was reported that following repeated intravenous use or topical ocular application, dendrimers with cationic end groups are often toxic, whereas anionic dendrimers are not. Thus, in order to reduce toxicity, it is necessary to modify the surface amine groups of these dendrimers with neutral or anionic moieties [35–37]. Recently, several studies have been published to report that ocular dendrimeric formulations were developed without cytotoxicity or irritation [38, 39]. For ocular drug delivery, it is very important to make sure that the dendrimers are safe because serious side effects may occur due to cytotoxicity at the ocular tissues. Safe dendrimer formulations for ocular drug delivery should have properties such as biocompatibility and low immunogenicity; thus they should be carefully designed and evaluated. Furthermore, in order to overcome the potential toxicity of the dendrimers, it is very important for ophthalmologists to participate and contribute to the scientific process alongside with chemists, formulation scientists and engineers.

## 4. Types of Dendrimers

**Figure 3:**
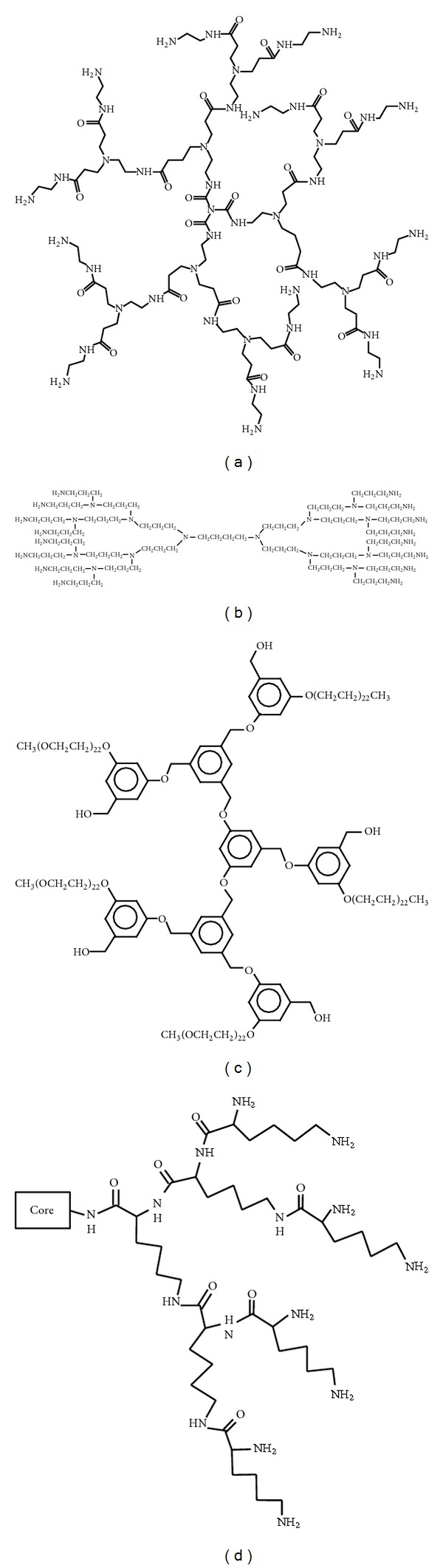
Structures of various types of dendrimers: (a) PAMAM dendrimer, (b) polypropyleneimine dendrimer, (c) polyaryl ether dendrimer, and (d) biodegradable polylysine dendron.

The first “dendrimer family” that was synthesized, characterized, and commercialized was poly(amidoamine) (PAMAM) dendrimers (Figure 3(a)) which were synthesized by the “divergent” method [25]. The structure of PAMAM dendrimers starts from an ammonia (NH_3_) or ethylenediamine (C_2_H_8_N_2_) molecule as a core that binds to the amine groups of branches (R-NH_2_) and amide (–CONH_2_R). Dendrimers growth reaches a critical point where the branching arms limit their development into higher generations due to steric effect that starts with G7. This effect decreases the synthetic yields of generations between G7 and G10 and prohibits the synthesis of any larger dendrimers [40, 41]. PAMAM dendrimers have a size range between 1.1 and 12.4 nm as their generations grow through 1–10 [42]. These dimensions have been compared to proteins (3–8 nm), linear polymer-drug conjugates (5–20 nm), and viruses (25–240 nm). Overall, PAMAM dendrimers are considered as ideal carriers for drug delivery due to their high aqueous solubility, large variety of surface groups, and unique architecture. For medical applications, the most widely studied PAMAM dendrimers have been the derivatives with an −NH_2_ surface, a −COOH surface, and an –OH surface [43].

Poly(amidoamine) organosilicon (PAMAMOS) dendrimers are silicon containing first commercial dendrimers which are inverted unimolecular micelles that contain exterior hydrophobic organosilicon and interiorly hydrophilic, nucleophilic polyamidoamine [44].

Polypropyleneimine (PPI) dendrimers (Figure 3(b)) have been investigated for their medical applications, but it has been shown that the presence of multiple cationic amine groups causes significant toxicity. These dendrimers are generally poly-alkyl amines with primary amine end groups and they are commercially available up to generation 5 [45]. Polyaryl ether dendrimers (Figure 3(c)) also have been evaluated for drug delivery, but it was found that it is required to use solubilizing groups at the periphery of them due to their poor water solubility [46].

In addition, biodegradable dendrimers have been designed such as those based on polylysine (Figure 3(d)), poly(disulfide amine), polyether, or polyester and after suitable surface modifications they have been developed as promising antiviral, antibacterial, chemotherapeutic, and vaccine carrier candidates. Glycodendrimers, that include carbohydrates in their architecture, also have great potential as drug carriers. Most of the glycodendrimers have saccharide residues on their outer surface, but glycodendrimers with a sugar central core have also been described [47]. Amino acid-based dendrimers, peptide dendrimers, hydrophobic dendrimers, and asymmetric dendrimers were also investigated for a variety of pharmaceutical applications [30, 48].

Surface engineered dendrimers were developed as a strategy for abatement of dendrimer toxicity. Functionalization helps the dendrimers gain some beneficial properties for their use as a drug delivery system as well as reducing the inherent toxicity [37]. One of the most popular modifications of dendrimer surface is PEGylation which offers so many advantages in addition to cytotoxicity reduction such as improved bioavailability/oral delivery application related to improved biodistribution and pharmacokinetics, enhanced solubility, increase in drug loading, sustained and controlled delivery of drugs, better transfection efficiency, and tumor localization [49]. Acetylation is another surface engineering approach to reduce toxicity based on modification of surface amino groups with acetyl groups [50].

There are already several dendrimer-based FDA-approved products in the market. For example, Stratus CS Acute Care (Dade Behring), containing dendrimer-linked monoclonal antibody, was launched for “cardiac diagnostic testing,” while another product based on modified “Tomalia-type PAMAM” dendrimers, SuperFect (Qiagen), is a well-known gene transfection agent available for a wide range of cell lines [51, 52]. In addition, VivaGel, which is a formulation of “polyanionic lysine G4 dendrimers” with an anionic surface of “naphthalenedisulfonate (SPL7013) in a Carbopol gel” that shows antiviral activity against HIV and HSV for the treatment, has already been taken into clinical trials by Starpharma, according to FDA requirements. It is currently in Phase III clinical trials and it is also the subject of a license agreement with Durex condoms for use as a condom coating [53, 54].

## 5. Interactions between Dendrimers and Drug Molecules

The dendrimer end group functionality can be modified to obtain molecules with novel biological properties such as cooperative receptor-ligand interactions, which will help dendrimers to interact with poorly soluble drugs. Dendrimers are able to increase the cellular uptake, bioavailability, and therapeutic efficacy, and they can also be used to optimize the biodistribution and to reduce the systemic toxicity, clearance, and degradation rate drugs [55].

**Figure 4:**
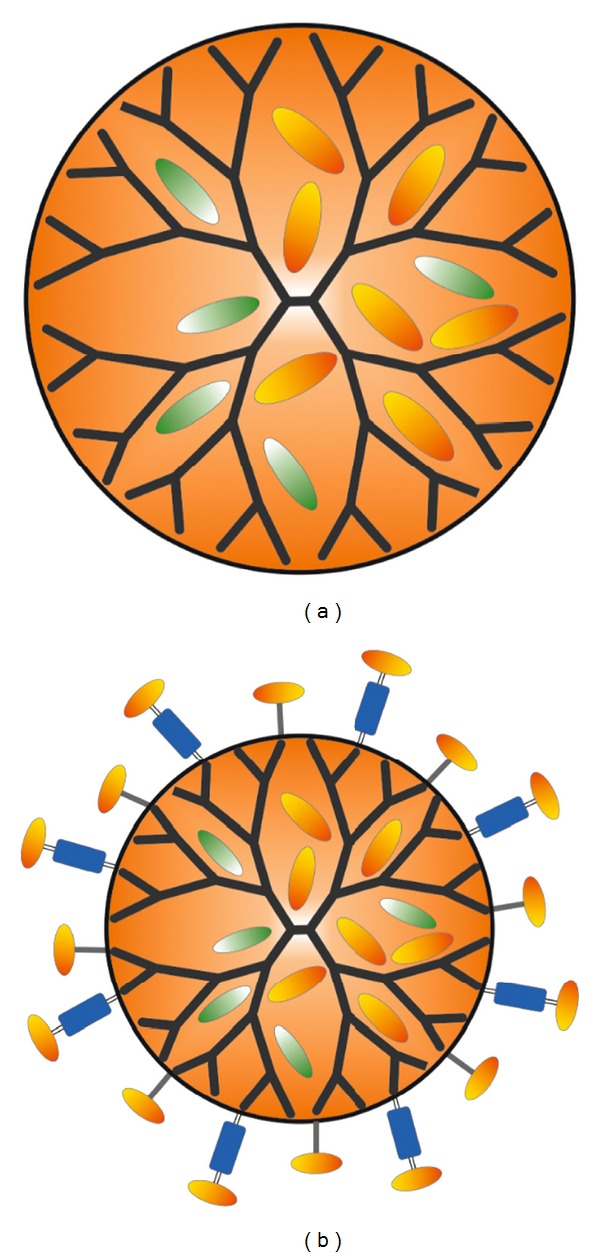
Schematic representation of drug encapsulated (a) and drug conjugated dendrimers (b).

There are two methods of dendrimer drug delivery: either lipophilic drugs can be encapsulated within the hydrophobic dendrimer cavity to make them water soluble or drugs can be covalently attached onto the dendrimer surface. Encapsulation traps the drug inside the dendrimer using the interaction between drug and the dendrimer or the steric bulk of the exterior of the dendrimer. The nature of drug encapsulation may be either a simple physical entrapment or can involve nonbonding-specific interactions within the dendrimer. On the other hand, the drug is attached to the exterior of the dendrimer in dendrimer/drug conjugates. Conjugates are usually prodrugs that are either inactive or have decreased activity. The covalent conjugation of the drugs was mainly used for targeting and achieving the higher drug payload, whereas the noncovalent interactions have resulted in higher solubility of insoluble drugs [23, 56]. A basic schematic representation of drug encapsulated and drug conjugated dendrimers is given in Figure 4. The unique architectural feature of dendrimers and dendrons makes them also ideal for the construction of cross-linked covalent gels, and for the self-assembled noncovalent gels [57].

Drug dendrimer interactions are affected by the structure, generation, concentration, and surface engineering of the dendrimers. For example, PAMAM and PPI have a slightly different dendritic framework. This difference in the internal branches makes PPI dendrimers relatively more hydrophobic compared to PAMAM dendrimers and that results in different solubilizing power [58]. Furthermore, modification of dendrimer surface can improve the therapeutic efficacy of drugs in terms of targeting and reduced toxicity.

### 5.1. Encapsulation of Drugs within Dendritic Structure

The acid-base reaction between the dendrimers and the guest molecules such as drugs with coulomb attractions pulls the guest molecules inside the dendrimer structure, whereas the hydrogen bonding keeps them together.

Jansen and coworkers reported the first encapsulation of a dye inside a dendrimer in 1994, the so-called “dendritic box” [59]. Guest molecules could be entrapped in the dendritic cavities during the synthetic process, with the help of a shell preventing diffusion from the structures, even after prolonged heating, sonication, or solvent extraction [60, 61]. Following encapsulation of dyes to dendrimers, anticancer drug encapsulation was the focus of the research. Kojima et al. encapsulated the anticancer drugs methotrexate and doxorubicin using G3 and G4 ethylenediamine-based poly(amidoamine) (PAMAM) dendrimers with poly(ethyleneglycol) monomethyl ether (M-PEG) grafts [62]. The same group also attached methotrexate and folic acid to the exterior of the dendritic structure and targeted the tumor cells using drug-dendrimer conjugates [63].

Dendrimers with an apolar core and polar shell have been referred to as “unimolecular micelles,” whereas the dendritic structure is independent of dendrimer concentration unlike conventional micelles [64]. However, this approach has a disadvantage that it is difficult to control the release of drugs from the dendrimer core. Poly(ethylene glycol) (PEG) has been used to modify dendrimers by conjugating PEG to the dendrimer surface to form a unimolecular micelle by providing a hydrophilic shell around the dendritic core. PEGylated dendrimers are of particular interest in drug delivery because they are biocompatible and highly water soluble and they have ability to modify the biodistribution of carrier systems [65, 66].

Zimmerman et al. synthesized “cored dendrimers” with modified dendritic architecture to encapsulate the drug. Following the synthesis, the core was removed via cleavage of ester bonds, while the rest of the structure remained the same as a consequence of robust ether linkages [67, 68].

“Dendrimeric block copolymers” have been synthesized with linear hydrophilic blocks and a hydrophobic dendritic block and their ability to complex “poorly water soluble” molecules have been investigated. A series of “G1–G5 PAMAM-block-PEG-block-PAMAM triblock copolymers” were synthesized by Kim et al. and studied as potential polymeric gene carrier [69].

Dendrimer-mediated complexation has advantages in terms of stability, release control, high drug loading, and lower toxicity of entrapped drugs. However, the noncovalent complexation often leads to lower drug encapsulation and complex stability compared to covalent conjugation [70].

### 5.2. Dendrimer Drug Conjugation

The outer surfaces of dendrimers have been investigated as potential interaction sites with drugs to increase the loading capacity. The number of available surface groups for drug interactions increases in twofolds with each higher generation of dendrimer. Drugs can be conjugated to dendritic systems through ester, amide, or some other linkage depending on dendritic surface, which can be hydrolysed by endosomal or lysosomal enzymes, inside the cell [55, 56]. There are several ionizable groups on the surface of dendrimers, where ionizable drugs can attach electrostatically. The main used points of attachment to conjugate drugs to dendrimers are amides, esters, disulfides, hydrazones, thiol-maleimide, and sulfinyl [43]. Many reports on drug loaded-dendrimers have shown that the release of the free drug can be enhanced by a suitable linker choice, especially, the linker/spacer length and flexibility. Some of the linkers are pH-sensitive and have proven to enhance intracellular release of the free drug [71].

Patri et al. compared the covalently conjugated drug and noncovalent inclusion complex in terms of release kinetics and efficacy, using generation 5 PAMAM dendrimers for targeting methotrexate. This study indicated that the inclusion complex releases the drug immediately and drug is active *in vitro*, while covalently conjugated drug is better suited for specifically targeted drug delivery [72]. A greater control over drug release can be achieved by the covalent drug attachment using biodegradable linkages than electrostatic drugs-dendrimer complexes. However, the major disadvantage of the conjugation is the possibility of less active drug release or too slow drug liberation potential to be effective *in vivo* [73].

### 5.3. Dendritic Gels

Pharmaceutical applications of hydrogels have attracted attention because of their architectural properties, drug loading capacity, and controlled drug release capability. Hydrogels are hydrophilic and “three-dimensional polymeric networks” have found application in drug delivery due to their high water absorbing capacity [74]. “*In situ* forming gels” have been investigated for a variety of applications including ocular, oral, nasal, vaginal, rectal, and injectable. [75, 76]. Dendrimers and dendrons' can be prepared with controlled molecular sizes due to their dendritic structure and their nature is between traditional gel polymers and the organic compounds with low molecular weight used in the “self assembled supramolecular gels” [57].

A “polymer network” is usually obtained with the use of a crosslinker during polymerization. Synthesis of hydrogels has been a function of the multivalent crosslinker behavior of dendritic molecules [77]. It has been shown that the dendritic branching has an important role in the self-assembly control. Furthermore, the repeated use of its key structural motifs can lead to multiple interactions between branched units and strengthen the noncovalent interactions responsible for the “self-assembly process” [78].

## 6. Ocular Applications of Dendrimeric Drug Delivery Systems

Dendrimers have been investigated for ophthalmic drug delivery since it offers a number of advantages as a carrier system. It has been reported that dendrimers were used for several purposes such as drug delivery, gene delivery, antioxidant delivery, peptide delivery, biomedical imaging, and genetic testing in ophthalmology [79]. A list of the ocular applications of dendrimers is given in Table 1.

Dendrimers are able to transport into and out of the cells. PAMAM dendrimers can have different cell entry pathways, depending on the functional groups on the surface. Anionic PAMAM dendrimers are endocytosed primarily through a caveolin-mediated process, whereas neutral and cationic dendrimers are internalizated in cells following a clathrin-mediated process. These pathways can be highly beneficial in the case of crossing the epithelial and retinal barriers in the cornea and retina [80, 81].

Different ocular application routes have been successfully used for dendrimeric drug delivery and better water solubility, permeability, bioavailability, and biocompatibility have been reported. Vandamme and Brobeck have evaluated several series of poly(amidoamine) (PAMAM) dendrimers for controlled ocular drug delivery and residence time of pilocarpine nitrate and tropicamide was found to be longer for the anionic dendrimer solutions. Results of a “miotic activity test” on albino rabbits showed that these PAMAM formulations improved pilocarpine nitrate bioavailability compared to the control and also prolonged the reduction of intraocular pressure (IOP), indicating increased precorneal residence time [82].

Durairaj et al. investigated dendrimeric polyguanidilyated translocators (DPTs), which are a class of dendrimers with tritolyl branches and surface guanidine groups as potential ophthalmic carriers for gatifloxacin, a “fourth generation fluoroquinolone” approved for conjunctivitis treatment. The results have indicated that the DPT forms stable gatifloxacin complexes and enhances solubility, permeability, anti-MRSA activity, and *in vivo* delivery of gatifloxacin and seems like a potential delivery system allowing once a day dosing [84].

“Phosphorus containing dendrimers,” with one quaternary ammonium salt as core and several carboxylic acid terminal groups have been synthesized from generation 0 to generation 2 by Spataro et al. These dendrimers have been tested *in vivo* in a rabbit model to evaluate ocular carteolol delivery and an increase of the carteolol amount in the aqueous humour is observed. No irritation was observed, even several hours after cationic dendrimers were applied [83].

Shaunak et al. have synthesized water soluble conjugates of D(+)-glucosamine and D(+)-glucosamine 6-sulfate with anionic PAMAM (G3.5) dendrimers to obtain synergistic “immunomodulatory and antiangiogenic effect.” When dendrimer glucosamine and dendrimer glucosamine 6-sulfate conjugates were used together in a clinically relevant scar tissue formation rabbit model after glaucoma filtration surgery, it has been shown that the long-term success of the surgery has increased from 30% to 80%. Furthermore, neither microbial infections nor clinical, biochemical, or hematological toxicity was observed in all animals [38].

Intraocular tumors such as retinoblastoma present a high risk of complications with high metastatic potential. One of the limited studies that have been done for drug delivery to intraocular tumors has explored the use of PAMAM dendrimers with carboxyl end groups (G3.5-COOH) for extended half life and sustained delivery of carboplatin with lowered therapeutic toxicity. Carboplatin-loaded PAMAM dendrimer complexes were explored in a transgenic murine retinoblastoma model, following subconjunctival administration. It was reported that the carboplatin-loaded dendrimer nanoparticles not only crossed the sclera, but were also retained for an extended period of time in the tumor vasculature, providing a sustained treatment effect [39].

In another research, biocompatible conjugates of lipophilic amino-acid dendrimers have been developed with collagen scaffolds to obtain better physical and mechanical properties and adhesion ability. Dendrimers-based approach was used for antivascular endothelial growth factor oligonucleotide (VEGF-ODN) delivery and successfully tested in a rat model to treat choroidal neovascularization (CNV). The results indicated that dendrimer/ODN-1 complexes significantly suppressed VEGF expression in cell level studies around 40 to 60%. Examinations of injected rat eyes also showed that no significant toxicity and damage were caused by the complex injections [85].

The repair of wounds such as corneal wounds that arise from surgical procedures has a significant importance for clinical aspects and research. Therefore, Duan and coworkers have generated highly crosslinked collagen using G2 polypropyleneimine octaamine dendrimers to use it as a tissue-engineering corneal scaffold. The optical transparencies of the dendrimer crosslinked collagen, EDC (1-ethyl-3-(3-dimethyl aminopropyl) carbodiimide hydrochloride), and glutaraldehyde crosslinked collagen thermal gels were compared and the transparency of dendrimer crosslinked collagen was found to be significantly higher. Results have shown that dendrimer crosslinked collagen gels improved “human corneal epithelial cell growth” and adhesion without cell toxicity [86]. The same group conjugated the “surface modified dendrimers” with cell adhesion peptides to be used as corneal tissue engineering scaffolds and the material has been incorporated into both bulk structures of the gels and onto the gel surface. Dendrimer amine groups were modified using carboxyl group and it was found that the surface modification promoted human corneal epithelial cell adhesion and proliferation [87].

Grinstaff has developed a series of dendrimeric adhesives, to repair corneal wounds, composed of generations 1, 2, and 3 (G1, G2, and G3) combined with PEG, glycerol, and succinic acid. The polymer was modified to contain terminal methacrylate groups, ([G1]-PGLSA-MA)_2_-PEG. Two strategies have been explored to form the ocular adhesives: a photocrosslinking reaction and a peptide ligation reaction to couple the individual dendrimers together. It was reported that both hydrogels were adhesive, elastic, soft, transparent, and hydrophilic. The *in vivo* studies in chicken eyes have indicated that the photocrosslinkable ([G1]-PGLSA-MA)_2_-PEG adhesive completely sealed on postoperative day. The histological studies have also demonstrated that wounds sealed using these adhesive gels appeared to be more complete after 28 days as compared to sutured wounds. The advantage of photo-cross-linked gels is the light-induced ability of the polymer to crosslink and adhere to the tissue; however, there is a potential risk of ocular damage when using light [88].

Photodynamic therapy is a potentially efficient treatment for retinoblastoma along with the other various solid tumours. Makky et al. have designed a photosensitizer, porphyrin-based glycodendrimers with the mannose-specific ligand protein Concanavalin A conjugated on to their surface, to specifically target the tumor cells in the retina. It was reported that the mannosylated dendrimers demonstrated specific interactions with the receptors in the lipid bilayer and accumulation in malignant ocular tissue was enhanced [90]. Dendrimers have also been explored as drug carriers and photosensitizers for exudative age-related macular degeneration (AMD) and CNV treatment. Nishiyama et al. investigated porphyrin-based dendrimers for their efficacy in treating retinal tumors and exudative AMD associated with CNV. The formulations showed selective accumulation within 24 h in the CNV lesions when injected into a CNV rat model [91, 92]. The same group developed phthalocyanine core-based dendrimer photosensitizers, which can be used to compact and deliver therapeutic genes with a targeting approach. Transgene expression was monitored only in the irradiated areas upon subconjunctival injection of the dendrimer formulation and followed by laser irradiation [93].

Sugisaki et al. investigated the accumulation of dendrimer porphyrin (DP), DP encapsulated polymeric micelles, and the efficacy of photodynamic therapy (PDT) using a corneal neovascularization model in mice. In this study a 3rd generation “aryl ether dendrimer zinc porphyrin” with carboxyl ending groups and polymeric micelles composed of the DP and PEG-poly(L-lysine) was used for PDT as a photosensitizer formulation. The results showed that following administration, in 1 hour to 24 hours, both DP and DP-micelle were accumulated in the neovascularized area [89].

Bravo-Osuna and coworkers investigated the *in vitro* and *in vivo* tolerance of carbosilane dendrimers (G1 and G3, anionic and cationic) for topical ophthalmic administration. Formulations were applied to New Zealand albino rabbits and it was reported that animals did not present either discomfort or clinical signs after the administration of dendrimer solutions. Nonionic interactions via hydrogen bonding between the PAMAM dendrimer surface moieties and mucins were observed. MTT test results also showed that anionic dendrimers were nontoxic for both conjunctival and corneal cells [94].

In a recent study, Puerarin–dendrimer complexes were prepared using PAMAM dendrimers (G3.5, G4, G4.5, and G5) and their physicochemical properties, *in vitro* release, corneal permeation, and ocular residence times were determined. Valia-Chien evaluated the corneal permeation and ocular residence time in rabbits using diffusion cells with excised corneas. It was reported that puerarin-dendrimer complexes exhibited longer residence time in rabbit eyes than puerarin eye drops, without damage to the corneal epithelium or endothelium. Also results of the *in vitro* release studies showed that puerarin release was much more slower from complexes than the free puerarin in PBS. However, corneal permeation studies suggested that there was no significant difference between puerarin-dendrimer complexes and puerarin eye drops on drug permeability coefficient [95].

Targeted drug therapy for retinal neuroinflammation was explored using “G4.0 hydroxyl-terminated PAMAM dendrimer-drug conjugate nanodevices” by Iezzi et al. Fluocinolone acetonide was conjugated to the dendrimers and *in vivo* efficacy was assessed for over a 4-week period, using the “Royal College of Surgeons rat retinal degeneration model.” It was reported that upon intravitreal administration PAMAM dendrimers were selectively localized within “activated outer retinal microglia” in two retinal degeneration rat models and the dendrimers were detected in the target cells, even 35 days after administration [96].

A PAMAM dendrimer hydrogel has been developed by Holden and coworkers that is made from “ultraviolet-cured PAMAM dendrimer” linked with PEG-acrylate chains for the delivery of two antiglaucoma drugs which were brimonidine (0.1% w/v) and timolol maleate (0.5% w/v). Dendrimeric hydrogel was obtained by crosslinking of the reactive acrylate groups, triggered by UV light. It was reported that the dendrimeric hydrogel was mucoadhesive and nontoxic to epithelial cells of human cornea. Higher uptake from “human corneal epithelial cells” and significantly enhanced “bovine corneal transport” were reported for both drugs, compared to the eye drops. The higher uptake in the dendrimeric hydrogel formulations explained the temporary decomposition of the corneal epithelial tight junctions [97]. The same group also developed a novel “hybrid PAMAM dendrimer hydrogel/poly(lactic-co-glycolic acid) (PLGA) nanoparticle platform (HDNP)”, again for codelivery of brimonidine and timolol maleate. The formulations were also evaluated in terms of their *in vitro* potential toxicity and it was found that the formulation was noncytotoxic to human corneal epithelial cells. Following topical administration of the HDNP in adult “normotensive Dutch-belted male rabbits,” formulation was found to be effective and maintained significantly higher concentrations of both drugs up to 7 days in aqueous humor and cornea compared to saline. Furthermore, it was reported that dendrimeric hydrogel and PLGA nanoparticles were not inducing any ocular inflammation or discomfort. This study demonstrated that this new formulation is able to enhance drug bioavailability, and following topical administration, it is capable of sustaining drug activity [98]. Wathier and coworkers also developed an *in situ* gel formulation using Lsy_x_Cys_y_ dendritic polymers to be used in cataract incisions instead of nylon sutures. It was reported that the hydrogel sealant procedure was simple and required less surgical time than conventional suturing and no additional tissue trauma was inflicted [99].

## 7. Conclusion

Effective treatment of ocular diseases is still a challenge in pharmaceutical research, because of the unique physiology of eye and presence of the ocular barriers especially in posterior segments of the eye. Research in ophthalmic drug delivery during the last two decades has undergone major advancements from the use of conventional formulations such as solutions, suspensions, and ointments to viscosity-enhancing *in situ* gel systems, different inserts, colloidal systems, and so forth.

Given their structural features, most of the ocular diseases would benefit from long-lasting drug delivery of dendrimers and dendrimer-based drug delivery systems. It was already reported that dendrimers present practical solutions to drug delivery issues such as solubility, biodistribution, and targeting. Since it is easy to control the features of dendrimers such as their size, shape, generation, branching length, molecular size, and surface functionality, these compounds are ideal carriers in pharmaceutical applications. Recent researches have shown that dendrimers are able to

- enhance the corneal residence time of drugs administered topically,
- target retinal neuroinflammation and provide targeted, sustained neuroprotection in retinal degeneration,
- deliver drugs to the retina upon systemic administration,
- be effective as corneal glues to potentially replace sutures following corneal surgeries.

Even though dendrimers are not approved yet for clinical use in the eye, their promising preclinical results can provide significant opportunities.

## Figures and Tables

**Table 1:** Ocular applications of dendrimers and dendrimeric delivery systems.

Drug	Dendrimer type	Administration	Treatment	Outcomes	References
Piocarpine nitrate and tropicamide	PAMAM G1.5-4	Topical	Myosis and mydriasis	Increased corneal residence and prolonged reduction of IOP	[82]
Carteolol	Phosphorus containing dendrimers	Topical	Glaucoma	Increased corneal residence and reduced toxicity and IOP	[83]
Gatifloxacin	Dendrimeric polyguanidilyated translocators	Topical	Conjunctivitis and intraocular infections	Enhanced corneal transport and increased antimicrobial activity	[84]
Glucosamine and glucosamine 6-sulfate	PAMAM G3.5-COOH	Subconjunctival injection	Antiangiogenic in glaucoma surgery	Reduced inflammation and no scar formation	[38]
Carboplatin	PAMAM G3.5-COOH (dendrimeric nanoparticles)	Subconjunctival injection	Retinoblastoma	Increased half life and bioavailability Reduced drug toxicity and tumor mass	[39]
VEGF-ODN	Lipophilic amino-acid dendrimer	Intravitreal injection	CNV	Prolonged suppression of VEGF and neovascularization	[85]
—	Polypropyleneimine octaamine G2	Corneal scaffold	Corneal tissue engineering	Enhanced human corneal epithelial cell growth	[86]
—	Surface modified-COOH ending dendrimers	Corneal scaffold	Corneal tissue engineering	Promoted adhesion and proliferation of human corneal epithelial cells	[87]
—	Modified G1, G2, and G3 dendrimers	Topical (corneal hydrogel adhesive)	Corneal wounds	Wound sealing and no scar formation	[88]
Photosensitizer	G3 aryl ether dendrimer zinc porphyrin	Intravenous injection-photodynamic theraphy	CNV	Accumulation in neovascularized area	[89]
Concanavalin A	Porphyrin glycodendrimers	Topical-Photodynamic therapy	Intraocular tumors and retinoblastoma	Enhanced targeting and reduced toxicity	[90]
—	Porphyrin dendrimers	Topical-Photodynamic therapy	AMD and CNV	Selective accumulation in inflammatory cells and prolonged retention time	[91, 92]
DNA	Phthalocyanine dendrimers			Accumulation in photo-irradiated areas and increased transgene expression	[93]
—	Anionicand cationic carbosilane dendrimers	Topical	Tolerance	Hydrogen bonding between mucin and PAMAM-enhanced retention time	[94]
Puerarin	PAMAM	Topical	Ocular hypertension and cataract	Increased bioavailability	[95]
Fluocinolone acetonide	PAMAM G4-OH	Intravitreal injection	Retinal neuroinflammation	Reduced inflammation	[96]
Brimonidine and timolol maleate	PAMAM hydrogel (G3)	Topical	Glaucoma	Increased uptake	[97]
Brimonidine and timolol maleate	Hybrid PAMAM dendrimer hydrogel/PLGA nanoparticle	Topical	Glaucoma	Increased uptake	[98]
—	Lys_x_Cys_y_ dendritic polymers-*in situ* gel	Topical	Cataract incisions	Wound sealing	[99]

## References

[1] Nagarwal RC, Kant S, Singh PN, Maiti P, Pandit JK (2009). Polymeric nanoparticulate system: a potential approach for ocular drug delivery. *Journal of Controlled Release*.

[2] Gaudana R, Jwala J, Boddu SHS, Mitra AK (2009). Recent perspectives in ocular drug delivery. *Pharmaceutical Research*.

[3] Lang JC (1995). Ocular drug delivery conventional ocular formulations. *Advanced Drug Delivery Reviews*.

[4] Ranta VP, Mannermaa E, Lummepuro K (2010). Barrier analysis of periocular drug delivery to the posterior segment. *Journal of Controlled Release*.

[5] Eljarrat-Binstock E, Pe'er J, Domb AJ (2010). New techniques for drug delivery to the posterior eye segment. *Pharmaceutical Research*.

[6] Del Amo EM, Urtti A (2008). Current and future ophthalmic drug delivery systems: a shift to the posterior segment. *Drug Discovery Today*.

[7] Ranta VP, Urtti A (2006). Transscleral drug delivery to the posterior eye: prospects of pharmacokinetic modeling. *Advanced Drug Delivery Reviews*.

[8] Quintana A, Raczka E, Piehler L (2002). Design and function of a dendrimer-based therapeutic nanodevice targeted to tumor cells through the folate receptor. *Pharmaceutical Research*.

[9] Diebold Y, Calonge M (2010). Applications of nanoparticles in ophthalmology. *Progress in Retinal and Eye Research*.

[10] Yasukawa T, Ogura Y, Tabata Y, Kimura H, Wiedemann P, Honda Y (2004). Drug delivery systems for vitreoretinal diseases. *Progress in Retinal and Eye Research*.

[11] Campbell M, Nguyen ATH, Kiang AS (2010). Reversible and size-selective opening of the inner blood-retina barrier: a novel therapeutic strategy. *Retinal Degenerative Diseases: Laboratory and Therapeutic Investigations*.

[12] Pahuja P, Arora S, Pawar P (2012). Ocular drug delivery system: a reference to natural polymers. *Expert Opinion on Drug Delivery*.

[13] Wadhwa S, Paliwal R, Paliwal SR, Vyas SP (2009). Nanocarriers in ocular drug delivery: an update review. *Current Pharmaceutical Design*.

[14] Raghava S, Hammond M, Kompella UB (2004). Periocular routes for retinal drug delivery. *Expert Opinion on Drug Delivery*.

[15] Patel PB, Shastri DH, Shelat PK, Shukla AK (2010). Ophthalmic drug delivery system: challenges and approaches. *Systematic Reviews in Pharmacy*.

[16] Perry HD, Solomon R, Donnenfeld ED (2008). Evaluation of topical cyclosporine for the treatment of dry eye disease. *Archives of Ophthalmology*.

[17] Yavuz B, Bozdağ Pehlıvan S, Ünlü N (2012). An overview on dry eye treartment: approaches for cyclosporin a delivery. *The Scientific World Journal*.

[18] Venkata Ratnam G, Madhavi S, Rajesh P (2011). Ocular drug delivery: an update review. *International Journal of Pharma and Bio Sciences*.

[19] Hamidi M, Azadi A, Rafiei P (2008). Hydrogel nanoparticles in drug delivery. *Advanced Drug Delivery Reviews*.

[20] Jiang J, Moore JS, Edelhauser HF, Prausnitz MR (2009). Intrascleral drug delivery to the eye using hollow microneedles. *Pharmaceutical Research*.

[21] Urtti A (2006). Challenges and obstacles of ocular pharmacokinetics and drug delivery. *Advanced Drug Delivery Reviews*.

[22] Sahoo SK, Diinawaz F, Krishnakumar S (2008). Nanotechnology in ocular drug delivery. *Drug Discovery Today*.

[23] Gillies ER, Frechet JM (2005). Dendrimers and dendritic polymers in drug delivery. *Drug Discovery Today*.

[24] Cheng YY, Xu ZH, Ma ML, Xu TW (2008). Dendrimers as drug carriers: applications in different routes of drug administration. *Journal of Pharmaceutical Sciences*.

[25] Esfand R, Tomalia DA (2001). Poly(amidoamine) (pamam) dendrimers: from biomimicry to drug delivery and biomedical applications. *Drug Discovery Today*.

[26] Hecht S, Frechet JMJ (2001). Dendritic encapsulation of function: applying nature's site isolation principle from biomimetics to materials science. *Angewandte Chemie International Edition*.

[27] Trivedi V, Patel U, Bhimani B, Daslania D, Patel G, Vyas B (2012). Dendrimer: a polymer of 21st century. *IJPRBS Journal*.

[28] Hodge P (1993). Organic-chemistry: polymer science branches out. *Nature*.

[29] Klajnert B, Bryszewska M (2001). Dendrimers: properties and applications. *Acta Biochimica Polonica*.

[30] Nanjwade BK, Bechra HM, Derkar GK, Manvi FV, Nanjwade VK (2009). Dendrimers: emerging polymers for drug-delivery systems. *European Journal of Pharmaceutical Sciences*.

[31] Tomalia DA (2005). Birth of a new macromolecular architecture: dendrimers as quantized building blocks for nanoscale synthetic polymer chemistry. *Progress in Polymer Science*.

[32] Bharti JP, Prajapati SK, Jaiswal MK, Yadav RD (2011). Dendrimer multifunctional nano-device: a review. *International Journal of Pharmaceutical Sciences and Research*.

[33] Garg T, Singh O, Arora S, Murthy R (2011). Dendrimer: a novel scaffold for drug delivery. *International Journal of Pharmaceutical Sciences Review and Research*.

[34] Kaminskas LM, Boyd BJ, Porter CJH (2011). Dendrimer pharmacokinetics: the effect of size, structure and surface characteristics on adme properties. *Nanomedicine*.

[35] Jevprasesphant R, Penny J, Attwood D, McKeown NB, D'Emanuele A (2003). Engineering of dendrimer surfaces to enhance transepithelial transport and reduce cytotoxicity. *Pharmaceutical Research*.

[36] Chen HT, Neerman MF, Parrish AR, Simanek EE (2004). Cytotoxicity, hemolysis, and acute in vivo toxicity of dendrimers based on melamine, candidate vehicles for drug delivery. *Journal of the American Chemical Society*.

[37] Jain K, Kesharwani P, Gupta U, Jain NK (2010). Dendrimer toxicity: let's meet the challenge. *International Journal of Pharmaceutics*.

[38] Shaunak S, Thomas S, Gianasi E (2004). Polyvalent dendrimer glucosamine conjugates prevent scar tissue formation. *Nature Biotechnology*.

[39] Kang SJ, Durairaj C, Kompella UB, O'Brien JM, Grossniklaus HE (2009). Subconjunctival nanoparticle carboplatin in the treatment of murine retinoblastoma. *Archives of Ophthalmology*.

[40] Eichman JD, Bielinska AU, Kukowska-Latallo JF, Baker JR (2000). The use of pamam dendrimers in the efficient transfer of genetic material into cells. *Pharmaceutical Science and Technology Today*.

[41] Silva NP, Menacho FP, Chorilli M (2012). Dendrimers as potential platform in nanotechnology-based drug delivery systems. *IOSR Journal of Pharmacy*.

[42] Tomalia DA (1990). Starburst dendrimers: molecular-level control of size, shape, surface chemistry, topology, and flexibility from atoms to macroscopic matter. *Angewandte Chemie International Edition*.

[43] Duncan R, Izzo L (2005). Dendrimer biocompatibility and toxicity. *Advanced Drug Delivery Reviews*.

[44] Malik A, Chaudhary S, Garg G, Tomar A (2012). Dendrimers: a tool for drug delivery. *Advances in Biological Research*.

[45] Malik N, Wiwattanapatapee R, Klopsch R (2000). Dendrimers: relationship between structure and biocompatibility in vitro and preliminary studies on the biodistribution of i-125-labelled polyamidoamine dendrimers in vivo. *Journal of Controlled Release*.

[46] Liu M, Kono K, Frechet JM (2000). Water-soluble dendritic unimolecular micelles: their potential as drug delivery agents. *Journal of Controlled Release*.

[47] Oliveira JM, Salgado AJ, Sousa N, Mano JF, Reis RL (2010). Dendrimers and derivatives as a potential therapeutic tool in regenerative medicine strategies: a review. *Progress in Polymer Science*.

[48] Medina SH, El-Sayed ME (2009). Dendrimers as carriers for delivery of chemotherapeutic agents. *Chemical Reviews*.

[49] Gajbhiye V, Vijayaraj Kumar P, Tekade RK, Jain NK (2009). Pegylated ppi dendritic architectures for sustained delivery of h2 receptor antagonist. *European Journal of Medicinal Chemistry*.

[50] Stasko NA, Johnson CB, Schoenfisch MH, Johnson TA, Holmuhamedov EL (2007). Cytotoxicity of polypropylenimine dendrimer conjugates on cultured endothelial cells. *Biomacromolecules*.

[51] Kumar P, Meena KP, Kumar P, Choudhary C, Thakur DS, Bajpayee P (2010). Dendrimer: a novel polymer for drug delivery. *JITPS Journal*.

[52] Menjoge AR, Kannan RM, Tomalia DA (2010). Dendrimer-based drug and imaging conjugates: design considerations for nanomedical applications. *Drug Discovery Today*.

[53] McCarthy TD, Karellas P, Henderson SA (2005). Dendrimers as drugs: discovery and preclinical and clinical development of dendrimer-based microbicides for hiv and sti prevention. *Molecular Pharmaceutics*.

[54] Wijagkanalan W, Kawakami S, Hashida M (2011). Designing dendrimers for drug delivery and imaging: pharmacokinetic considerations. *Pharmaceutical Research*.

[55] D'Emanuele A, Attwood D (2005). Dendrimer-drug interactions. *Advanced Drug Delivery Reviews*.

[56] Jain NK, Gupta U (2008). Application of dendrimer-drug complexation in the enhancement of drug solubility and bioavailability. *Expert Opinion on Drug Metabolism and Toxicology*.

[57] Smith DK (2006). Dendritic gels: many arms make light work. *Advanced Materials*.

[58] Richter-Egger DL, Tesfai A, Tucker SA (2001). Spectroscopic investigations of poly(propyleneimine)dendrimers using the solvatochromic probe phenol blue and comparisons to poly(amidoamine) dendrimers. *Analytical Chemistry*.

[59] Jansen JFGA, Debrabandervandenberg EMM, Meijer EW (1994). Encapsulation of guest molecules into a dendritic box. *Science*.

[60] Jansen JFGA JFGA, Debrabandervandenberg EMM, Meijer EW (1995). The dendritic box and bengal rose. *Polymeric Materials*.

[61] Jansen JFGA, Meijer EW, Debrabandervandenberg EMM (1995). The dendritic box: shape-selective liberation of encapsulated guests. *Journal of the American Chemical Society*.

[62] Kojima C, Kono K, Maruyama K, Takagishi T (2000). Synthesis of polyamidoamine dendrimers having poly(ethylene glycol) grafts and their ability to encapsulate anticancer drugs. *Bioconjugate Chemistry*.

[63] Kono K, Liu M, Frechet JMJ (1999). Design of dendritic macromolecules containing folate or methotrexate residues. *Bioconjugate Chemistry*.

[64] Stevelmans S, vanHest JCM, Jansen JFGA, vanBoxtel DAFJ, Vanden Berg EMMD, Meijer EW (1996). Synthesis, characterization and guest-host properties of inverted unimolecular dendritic micelles. *Journal of the American Chemical Society*.

[65] Pan GF, Lemmouchi Y, Akala EO, Bakare O (2005). Studies on pegylated and drug-loaded pamam dendrimers. *Journal of Bioactive and Compatible Polymers*.

[66] Greenwald RB, Choe YH, McGuire J, Conover CD (2003). Effective drug delivery by pegylated drug conjugates. *Advanced Drug Delivery Reviews*.

[67] Wendland MS, Zimmerman SC (1999). Synthesis of cored dendrimers. *Journal of the American Chemical Society*.

[68] Schultz LG, Zhao Y, Zimmerman SC (2001). Synthesis of cored dendrimers with internal cross-links. *Angewandte Chemie International Edition*.

[69] Kim T, Seo HJ, Choi JS (2004). Pamam-peg-pamam: novel triblock copolymer as a biocompatible and efficient gene delivery carrier. *Biomacromolecules*.

[70] Boas U, Heegaard PM (2004). Dendrimers in drug research. *Chemical Society Reviews*.

[71] El Kazzouli S, Mignani S, Bousmina M, Majoral JP (2012). Dendrimer therapeutics: covalent and ionic attachments. *New Journal of Chemistry*.

[72] Patri AK, Kukowska-Latallo JF, Baker JR (2005). Targeted drug delivery with dendrimers: comparison of the release kinetics of covalently conjugated drug and non-covalent drug inclusion complex. *Advanced Drug Delivery Reviews*.

[73] Kaminskas LM, McLeod VM, Porter CJ, Boyd BJ (2012). Association of chemotherapeutic drugs with dendrimer nanocarriers: an assessment of the merits of covalent conjugation compared to noncovalent encapsulation. *Molecular Pharmaceutics*.

[74] Hoare TR, Kohane DS (2008). Hydrogels in drug delivery: progress and challenges. *Polymer*.

[75] Nirmal HB, Bakliwal SR, Pawar SP (2010). In-situ gel: new trends in controlled and sustained drug delivery system. *International Journal of PharmTech Research*.

[76] Navath RS, Menjoge AR, Dai H, Romero R, Kannan S, Kannan RM (2011). Injectable pamam dendrimer-peg hydrogels for the treatment of genital infections: formulation and in vitro and in vivo evaluation. *Molecular Pharmaceutics*.

[77] Sontjens SHM, Nettles DL, Carnahan MA, Setton LA, Grinstaff MW (2006). Biodendrimer-based hydrogel scaffolds for cartilage tissue repair. *Biomacromolecules*.

[78] Jang WD, Aida T (2003). Dendritic physical gels: structural parameters for gelation with peptide-core dendrimers. *Macromolecules*.

[79] Marco Zarbin A, James Leary F, Montemagno C, Ritch R, Humayun MS, Ryan SJ (2013). Nanomedicine in ophthalmology. *Retina*.

[80] Perumal OP, Inapagolla R, Kannan S, Kannan RM (2008). The effect of surface functionality on cellular trafficking of dendrimers. *Biomaterials*.

[81] Kambhampati SP, Kannan RM (2013). Dendrimer nanoparticles for ocular drug delivery. *Journal of Ocular Pharmacology and Therapeutics*.

[82] Vandamme TF, Brobeck L (2005). Poly(amidoamine) dendrimers as ophthalmic vehicles for ocular delivery of pilocarpine nitrate and tropicamide. *Journal of Controlled Release*.

[83] Spataro GG, Malecaze F, Turrin C-O (2010). Designing dendrimers for ocular drug delivery. *European Journal of Medicinal Chemistry*.

[84] Durairaj C, Kadam RS, Chandler JW, Hutcherson SL, Kompella UB (2010). Nanosized dendritic polyguanidilyated translocators for enhanced solubility, permeability and delivery of gatifloxacin. *Investigative Ophthalmology and Visual Science*.

[85] Marano RJ, Toth I, Wimmer N, Brankov M, Rakoczy PE (2005). Dendrimer delivery of an anti-vegf oligonucleotide into the eye: a long-term study into inhibition of laser-induced cnv, distribution, uptake and toxicity. *Gene Therapy*.

[86] Duan X, Sheardown H (2006). Dendrimer crosslinked collagen as a corneal tissue engineering scaffold: mechanical properties and corneal epithelial cell interactions. *Biomaterials*.

[87] Duan XD, McLaughlin C, Griffith M, Sheardown H (2007). Biofunctionalization of collagen for improved biological response: scaffolds for corneal tissue engineering. *Biomaterials*.

[88] Grinstaff MW (2007). Designing hydrogel adhesives for corneal wound repair. *Biomaterials*.

[89] Sugisaki K, Usui T, Nishiyama N (2008). Photodynamic therapy for corneal neovascularization using polymeric micelles encapsulating dendrimer porphyrins. *Investigative Ophthalmology and Visual Science*.

[90] Makky A, Michel JP, Maillard P, Rosilio V (2011). Biomimetic liposomes and planar supported bilayers for the assessment of glycodendrimeric porphyrins interaction with an immobilized lectin. *Biochimica et Biophysica Acta*.

[91] Nishiyama N, Stapert HR, Zhang GD (2003). Light-harvesting ionic dendrimer porphyrins as new photosensitizers for photodynamic therapy. *Bioconjugate Chemistry*.

[92] Nishiyama N, Morimoto Y, Jang WD, Kataoka K (2009). Design and development of dendrimer photosensitizer-incorporated polymeric micelles for enhanced photodynamic therapy. *Advanced Drug Delivery Reviews*.

[93] Nishiyama N, Iriyama A, Jang WD (2005). Light-induced gene transfer from packaged DNA enveloped in a dendrimeric photosensitizer. *Nature Materials*.

[94] Bravo-Osuna I, Herrero-Vanrell R, Molina Martínez IT (2010). In vitro and in vivo tolerance studies of carbosilane dendrimers for ophthalmic administration. *Investigative Ophthalmology and Visual Science*.

[95] Yao WJ, Sun KX, Mu HJ (2010). Preparation and characterization of puerarindendrimer complexes as an ocular drug delivery system. *Drug Development and Industrial Pharmacy*.

[96] Iezzi R, Guru BR, Glybina IV, Mishra MK, Kennedy A, Kannan RM (2012). Dendrimer-based targeted intravitreal therapy for sustained attenuation of neuroinflammation in retinal degeneration. *Biomaterials*.

[97] Holden CA, Tyagi P, Thakur A (2012). Polyamidoamine dendrimer hydrogel for enhanced delivery of antiglaucoma drugs. *Nanomedicine*.

[98] Yang H, Tyagi P, Kadam RS, Holden CA, Kompella UB (2012). Hybrid dendrimer hydrogel/plga nanoparticle platform sustains drug delivery for one week and antiglaucoma effects for four days following one-time topical administration. *ACS Nano*.

[99] Wathier M, Jung PJ, Carnahan MA, Kim T, Grinstaff MW (2004). Dendritic macromers as in situ polymerizing biomaterials for securing cataract incisions. *Journal of the American Chemical Society*.

